# The Effects of Quantitative Trait Architecture on Detection Power in Short-Term Artificial Selection Experiments

**DOI:** 10.1534/g3.120.401287

**Published:** 2020-07-09

**Authors:** R. Nicolas Lou, Nina O. Therkildsen, Philipp W. Messer

**Affiliations:** * Department of Natural Resources, Cornell University, Ithaca, New York 14853; † Department of Computational Biology, Cornell University, Ithaca, New York 14853

**Keywords:** experimental evolution, molecular adaptation, forward simulation, power analysis, temporal data, detecting selection

## Abstract

Evolve and resequence (E&R) experiments, in which artificial selection is imposed on organisms in a controlled environment, are becoming an increasingly accessible tool for studying the genetic basis of adaptation. Previous work has assessed how different experimental design parameters affect the power to detect the quantitative trait loci (QTL) that underlie adaptive responses in such experiments, but so far there has been little exploration of how this power varies with the genetic architecture of the evolving traits. In this study, we use forward simulation to build a more realistic model of an E&R experiment in which a quantitative polygenic trait experiences a short, but strong, episode of truncation selection. We study the expected power for QTL detection in such an experiment and how this power is influenced by different aspects of trait architecture, including the number of QTL affecting the trait, their starting frequencies, effect sizes, clustering along a chromosome, dominance, and epistasis patterns. We show that all of these parameters can affect allele frequency dynamics at the QTL and linked loci in complex and often unintuitive ways, and thus influence our power to detect them. One consequence of this is that existing detection methods based on models of independent selective sweeps at individual QTL often have lower detection power than a simple measurement of allele frequency differences before and after selection. Our findings highlight the importance of taking trait architecture into account when designing and interpreting studies of molecular adaptation with temporal data. We provide a customizable modeling framework that will enable researchers to easily simulate E&R experiments with different trait architectures and parameters tuned to their specific study system, allowing for assessment of expected detection power and optimization of experimental design.

Artificial selection experiments can provide insights into the mechanisms that allow populations to adapt to strong selection pressures (Hill and Caballero 1992; Fuller *et al.* 2005; Garland and Rose 2009). When combined with population-level genome sequencing, such experiments can also help us elucidate the genetic architecture of the selected traits by revealing the quantitative trait loci (QTL) that underlie observed adaptive responses (Burke *et al.* 2010; Schlötterer *et al.* 2015). This rationale forms the basis of the evolve and resequence (E&R) method for QTL detection (Turner *et al.* 2011; Long *et al.* 2015), in which one seeks to identify the alleles that have systematically changed in frequency over the course of a selection experiment. Such E&R experiments have now been successfully performed in a wide range of study systems (*e.g.*, *Escherichia coli* (Barrick *et al.* 2009; Tenaillon *et al.* 2012), yeast (Parts *et al.* 2011; Lang *et al.* 2013), *Drosophila melanogaster* (Burke *et al.* 2010, Zhou *et al.* 2011, Turner *et al.* 2011), other *Drosophila* species (Seabra *et al.* 2018; Kelly and Hughes 2019), *Caenorhabditis* species (Teotónio *et al.* 2017), and mice (Chan *et al.* 2012; Castro *et al.* 2019)). For example, with E&R experiments, Tenaillon *et al.* (2012) uncovered 600 loci associated with high-temperature tolerance in *E. coli*, and Burke *et al.* (2010) identified several dozen genomic regions responding to selection for accelerated development in *D. melanogaster*.

In higher eukaryotes, practical constraints typically impose severe limits on the size of the experimental population and the number of generations an E&R experiment can be conducted for. Selection pressure is therefore typically kept high so that an adequate trait response can still be achieved. One consequence of such small population size and strong selection is that effective population sizes tend to be low in these experiments, resulting in high levels of genetic drift. In addition, because recombination will be less effective at breaking up linkage in a short experiment, there could be substantial hitchhiking of neutral alleles (Smith and Haigh 1974) as well as Hill-Robertson interference (Hill and Robertson 1966) between selected alleles. All of these factors can limit power and introduce false positives in E&R experiments (Kessner and Novembre 2015).

The first studies to assess the power of E&R experiments for QTL detection in higher eukaryotes used forward-in-time population simulations to model evolutionary dynamics at individual QTL (Kofler and Schlötterer 2014; Baldwin-Brown *et al.* 2014). These studies provided important insights into how detection power is affected by basic population genetics parameters such as recombination rate, linkage disequilibrium (LD), and the levels of neutral diversity in the regions surrounding the QTL. In addition, they explored how different aspects of the experimental design such as selection strength, population size, duration of the experiment, and number of experimental replicates can be tuned to maximize detection power.

However, two assumptions of these early studies have turned out to limit their generalizability: First, allele frequency dynamics at individual QTL were modeled as independent selective sweeps, parameterized by constant selection coefficients. Such models will often fail to capture key aspects of the evolutionary dynamics of QTL underlying polygenic traits, where an individual’s fitness is a function of its trait value instead of some constant selection coefficients at QTL (Burke *et al.* 2010; Kessner and Novembre 2015; Franssen *et al.* 2017). Second, these studies either focused on regions with high recombination (Kofler and Schlötterer 2014) or modeled only a single QTL (Baldwin-Brown *et al.* 2014). This tends to underrepresent the strong genome-wide pattern of linked selection on neutral loci typical for polygenic adaptation and effectively neglects the possibility of Hill-Robertson interference between QTL, which can affect the evolutionary dynamics in complex ways, thereby impinging on QTL detection power (Hill and Robertson 1966; Smith and Haigh 1974; Lang *et al.* 2013; Kessner and Novembre 2015). Thus, to more accurately describe polygenic trait evolution in E&R experiments, we need to adopt more realistic quantitative genetic models in which the selected trait is defined explicitly and the loci underlying the trait are modeled in the explicit context of a recombining chromosome.

The selection model introduced by Kessner and Novembre (2015) constitutes an important first step in this direction, but it assumed a limited set of genetic trait architectures in which only the number of QTL was variable. In reality, the traits of interest in E&R experiments could span a considerable variety of genetic architectures, and we typically know very little about this architecture for any given polygenic trait (Hansen 2006; Mackay *et al.* 2009; Gibson 2012). For example, in addition to how many QTL control a given trait, these QTL could be distributed uniformly along the chromosome, or they could cluster in certain regions. Effect sizes could be similar among the individual QTL, or they could vary according to some distribution. The frequencies of the selected alleles might be biased toward lower or toward higher frequencies, as compared to neutrally segregating alleles. The effects of these alleles on the trait might be recessive, dominant, or fall somewhere in between. Furthermore, there could be epistatic interactions of varying complexity among specific sets of QTL.

Some of these aspects of trait architecture have previously been demonstrated to affect the evolutionary dynamics of trait evolution in E&R experiments. Franssen *et al.* (2017), for example, showed that the effect size distribution and starting frequency of QTL can profoundly influence their frequency trajectories in response to selection. Similarly, Stetter *et al.* (2018) showed that the effect sizes of QTL are a key determinant of their frequencies at the end of a selection experiment. As a result, depending on their underlying genetic architecture, certain traits might generally be more suitable for QTL detection in E&R experiments, and for any given trait, there could be systematic biases in terms of which QTL will be more easily detected and which will be missed. Due to interactions among different factors, this is unlikely to only depend on effect size, so a power analysis considering broader aspects of trait architecture in E&R experiments is required to properly interpret results.

A second limitation in previous studies of detection power in E&R experiments is that they have focused primarily on insect populations like *Drosophila* (Kofler and Schlötterer 2014; Baldwin-Brown *et al.* 2014; Kessner and Novembre 2015), which are well-suited organisms for such experiments due to their short generation times, relative ease at which large populations can be reared, and rich genomic resources. However, for certain questions (*e.g.*, the genomic basis of vertebrate traits), selection experiments on larger and longer-living organisms may be necessary despite the additional logistical challenges. E&R experiments on such organisms will typically be restricted to fewer generations, therefore requiring even larger selection intensities to achieve measurable changes in trait value. The greater selection intensity could lead to characteristic differences in evolutionary dynamics compared to experiments carried out over larger numbers of generations and a potential decline in detection power. Nevertheless, many short-term selection experiments have been performed on such larger and longer-living species, even though in many cases their original intentions were not QTL detection (*e.g.*, mice (van Oortmerssen and Bakker 1981; Chan *et al.* 2012; Barrett *et al.* 2019), guppies (Houde 1994), silversides (Conover and Munch 2002; Therkildsen *et al.* 2019), voles (Sadowska *et al.* 2008), sticklebacks (Barrett *et al.* 2011), and zebrafish (Uusi-Heikkilä *et al.* 2017)). In addition, many common human practices, such as animal domestication and size-selective harvesting (through fishing and hunting), resemble E&R experiments in key aspects such as high selection pressure and specific traits targeted by selection (*e.g.*, domestication of salmonid fish (Christie *et al.* 2016, Gutierrez *et al.* 2016) and chicken (Rubin *et al.* 2010, Johansson *et al.* 2010, Fallahsharoudi *et al.* 2017) or size-selective harvesting in Atlantic cod (Swain *et al.* 2007, Therkildsen *et al.* 2013) and bighorn sheep (Coltman *et al.* 2003, Pigeon *et al.* 2016)). To illustrate the widespread short-term selection experiments in the literature, we have summarized a set of examples in Table S1 of the supplementary materials. With high-throughput sequencing becoming cheaper and more widely accessible, genomic data can now be obtained from such experiments and practices on a broad scale. This raises the question of how well time-series data collected over a small number of generations can help us illuminate the molecular basis of selected traits in these larger and longer-living species.

In this paper, we use forward genetic simulations to systematically assess how different aspects of trait architecture are expected to affect the evolutionary dynamics and power to detect QTL in E&R experiments. Loosely inspired by a size-selection experiment performed on the Atlantic silverside (*Menidia menidia*) to examine impacts of fisheries-induced evolution (Conover and Munch 2002; Therkildsen *et al.* 2019), our model setup is comparable to experimental designs applicable to E&R studies in larger and longer-living species in general (*e.g.*, Sadowska *et al.* 2008; Uusi-Heikkilä *et al.* 2017). The specific aspects of trait architecture that we investigate are the number of QTL contributing to a selected trait, the clustering of QTL along the chromosome, the effect size distribution among the QTL, the starting frequencies of the QTL, dominance, and epistasis patterns. We show that most of these variables can greatly influence QTL detection power, often in complex ways where the effect of one aspect of the architecture depends on other aspects of the architecture. We also demonstrate that although short-term selection experiments generally have limited power in detecting the full set of QTL contributing to the selected trait, some QTL with higher starting frequency and larger effect size can still be detected, suggesting that these experiments can be a useful approach for studying the genomic basis of adaptation in species with longer generation time. Motivated by these insights, we further discuss how optimal detection strategies, including detection methods and experimental design, may vary under different quantitative trait architectures.

## Methods

### Simulation of E&R experiments

We used forward genetic simulations to model E&R experiments in which divergent truncating selection is imposed on a quantitative trait over four consecutive generations. The analysis pipeline consists of the following stages:

1. Burn-in to create genetic variation in a starting population
2. Construction of QTL architecture
3. Selection on the trait
4. QTL detection
5. Power analysis

### Burn-in to generate genetic variation in the starting population

To model a population under mutation-drift equilibrium prior to the selection experiment, we first simulated a 30 Mbp-long chromosome evolving neutrally in a diploid population of *N* = 1,000 individuals for 10*N* generations. Although a population size of 1,000 may seem small for most natural populations, it is important to point out that because we are simulating neutral evolution during this phase, it is primarily the product *Nμ* that will determine patterns of neutral diversity, rather than the absolute value of *N*. We set the mutation rate to *μ* = 2×10^−8^, corresponding to an expected equilibrium level of nucleotide diversity of *π* = 4*Nμ* = 8×10^−5^. While this value of *π* is still comparatively small for many species, it was chosen for computational efficiency and we note that in our analyses of QTL detection power the value of *π* is only expected to affect the absolute number of false positives, but not the false positive rate (*i.e.*, the probability that any given neutral SNP is falsely detected as a QTL, also see Figure S1).

More critical to our power analysis is the rate of recombination, as it will determine the rate at which new allele combinations can arise during the selection experiments and also affect the amount of interference between QTL and the level of hitchhiking of neutral SNPs with selected alleles. We chose a rate of *r* = 1 cM/Mbp for our simulations. Under these parameters, linkage disequilibrium (LD), as measured by *r*^2^, decays to 0.5 over a distance of approximately 25 kbp in our simulated population (Sved 1971).

### Assignment of QTL and construction of the trait architecture

In our standard trait model, we randomly selected *n* of the existing neutral SNPs after the burn-in to become the QTL affecting the trait. We then randomly picked half of these SNPs to which we assigned a positive effect (+1) to the derived allele and a zero effect to the ancestral allele. For the other half of these selected SNPs, we assigned a negative effect (-1) to the derived allele and a zero effect to the ancestral allele. We note that in the truncating selection design that we will employ below, it is only the relative difference between the effect sizes of the derived and ancestral alleles at each QTL that matters for the selection response. For example, when we select for larger trait values in the experiment, the +1 derived alleles are selected for and the corresponding ancestral alleles are selected against, whereas the -1 derived alleles are selected against and the corresponding ancestral alleles are selected for. We further assumed an additive dominance relationship (*h* = 1/2) at individual QTL and also additive effects across QTL. Under this model, the average trait value in the population is expected to be zero at the start of a selection experiment and the distribution of trait values among individuals should be approximately Gaussian (as long as *n* is sufficiently large), consistent with many polygenic quantitative traits in nature (Mackay 2009). We did not model the effect of environmental factors on trait value (*i.e.*, the broad-sense heritability of the trait is set to a value of 1 in our simulations).

Although many aspects of this standard model are idealistic, our goal here is not to construct the most realistic trait model but to qualitatively evaluate how different parameters of the model can affect detection power. Six key parameters of this standard model were thus varied to explore different trait architectures: (i) the number of QTL, (ii) the clustering of QTL along the chromosome, (iii) the effect size distribution of QTL, (iv) the initial allele frequency distribution of the SNPs chosen to become QTL, (v) the dominance relationship between the ancestral and derived alleles, and (vi) the presence of epistatic interactions among pairs of QTL. The specific implementations of each of these modified models are described in the relevant sections below, and Table S2 gives a summary of all parameters of interest and their values that we have tested.

### Selection experiment on the trait

We modeled the selection experiment closely after the silverside experiment that served as a motivating example for this study (Conover and Munch 2002; Therkildsen *et al.* 2019). Specifically, the population is subjected to divergent truncating selection, generating two separate lines from the burn-in population: a high-trait-value line and a low-trait-value line. For the “high” line, we selected the 10% of individuals with the highest trait values in every generation to become the parents for the next generation (obtained by Wright-Fisher sampling). The “low” line was generated analogously by choosing the 10% of individuals with the lowest trait value as parents for the next generation. Population size was kept at 1,000 individuals in every generation in each line, and each line was run for four generations of truncating selection. Because of this short duration, the impact of new mutations occurring during the selection experiment should be negligible, and we therefore set the mutation rate to zero after the burn-in while recombination events continue to occur at a rate of 1 cM/Mb, which is similar in magnitude to the observed rates in many species of interest (*e.g.*, 0.63 cM/Mbp in mouse (Shifman *et al.* 2006), 0.97 cM/Mbp in dog (Wong *et al.* 2010), 1.5 cM/Mbp in zebra finch (Backström *et al.* 2010), 3.11 cM/Mbp in three-spined stickleback (Roesti *et al.* 2013)).

As noted in the introduction, this simulated experimental design is somewhat atypical among traditional E&R studies in that it lasts only four generations and does not have replicated populations. Because of logistical constraints, this is indeed the only feasible design with many larger and long-living study organisms, but here we also explored how detection power in our standard QTL model can improve if an experiment can be conducted for five more generations and if two or five experimental replicates are included in the experiment. To model experimental replication, we started with the same burn-in population at the first generation, constructed the same trait architecture for each experimental replicate, but simulated the selection experiment with a different random seed for each replicate. This therefore corresponds to a scenario where replicated populations are created after the first generation of the selection experiment. Results from these models are not central to the main objectives of the paper and will only be shown in the discussion section.

### QTL detection

In each generation, we took a random sample of 50 individuals and measured the allele frequencies of all SNPs in the sample. Following Kessner and Novembre (2015), we took the absolute values of sampled allele frequency differences between the last generations in the “high” and “low” lines at each SNP (denoted by *D*) as a summary statistic for QTL detection (*i.e.*, *D* = |*f_high_* - *f_low_*|, where *f_high_* is the sample allele frequency in the last generation of the “high” line and *f_low_* is the sampled allele frequency in the last generation of the “low” line). In addition, since alleles that start at intermediate frequencies tend to generate larger *D*-values simply due to genetic drift, we performed an angular transformation on the sample allele frequencies. We call the absolute values of the transformed allele frequency differences between the two lines “*transformed-D*” (*i.e.*, *transformed-D* = |2 × sin^-1^($\sqrt{\left(f_{\mathrm{high}}\right)}$) – 2 × sin^-1^($\sqrt{\left(f_{\mathrm{low}}\right)}$)| / π. The *transformed-D* summary statistic has the same scale of 0-1 as *D*, where 0 signifies no difference in allele frequency at the end of the two selection lines and 1 signifies alleles that are fixed in one selection line and lost in the other. Unlike *D*, the *transformed-D* statistic is independent of the starting allele frequency at neutral loci to a first approximation (Fisher and Ford 1947; Walsh and Lynch 2018; Kelly and Hughes 2019). When there are multiple experimental replicates involved, we took the average of *transformed-D* value (or *D* value where this was calculated) across these experimental replicates as the final summary statistics for each SNP.

Besides these simple and intuitive summary statistics based on allele frequency differences, several more sophisticated, model-based detection methods have been developed in recent years that can take advantage of the full allele frequency trajectory estimated across subsequent time points (Malaspinas 2016). After a comprehensive literature review, we selected two representatives of such methods for comparison with the simple *D* and *transformed-D* statistics: WFABC (Foll *et al.* 2015) and ApproxWF (Ferrer-Admetlla *et al.* 2016). We chose these two methods because they are widely used, require only allele frequency data, provide extensive documentation, and employ two contrasting approaches. Both of these methods are based on a classic selective sweep model parameterized by fixed selection coefficients. WFABC employs an approximate Bayesian computation framework, in which a large number of simulations are compared to identify the simulated datasets that are most similar to the actual data and compute posterior probabilities of selection coefficients (Foll *et al.* 2015). ApproxWF uses a “mean transition time approximation” to discretize the continuous diffusion process and infers the selection coefficient via a Bayesian approach (Ferrer-Admetlla *et al.* 2016).

After a series of tests, we adjusted some parameter values in WFABC from the default to optimize its detection power on our standard model, by increasing the number of simulated datasets to 1,000,000, lowering the acceptance rate to 0.00, and assigning a uniform prior to selection coefficients with an upper bound of 1 and lower bound of -1. With ApproxWF, we used its default Markov Chain Monte Carlo settings, with 10,000 iterations and 51 frequency states distributed on quadratic grid, and assigned a normal prior to selection coefficients, with mean of 0 and standard deviation of 0.1, truncated at 1 and -1. We also set a fixed dominance coefficient of 0.5 to reduce the computational complexity in ApproxWF.

Both methods output a posterior distribution for the selection coefficient for each SNP, and from this distribution, we calculated a mean selection coefficient *s* for each SNP, as well as a posterior probability *p* for *s* > 0, as recommended by authors of these methods (Foll *et al.* 2015, Ferrer-Admetlla *et al.* 2016). We applied WFABC and ApproxWF to the high-trait-value line and the low-trait-value line separately, and then took the average of *s* and *p* across the two lines while accounting for the directionality of selection (*i.e.*, $\underset{¯}{s}$ = (*s_high_* – *s_low_*) / 2, $\underset{¯}{p}$= (*p_high_* + (1 - *p_low_*)) / 2). Lastly, since +1 alleles and -1 alleles are not distinguished and both are considered as QTL in the power analysis, the signs of selection coefficients would not matter, so we took |$\underset{¯}{s}$| and |$\underset{¯}{p}$ - 0.5| as the final summary statistics for each SNP. After testing for the detection power of these statistics, we chose to use |$\underset{¯}{s}$| for WFABC and |$\underset{¯}{p}$ - 0.5| for ApproxWF to maximize their performance.

### Power analysis

We calculated receiver operating characteristic (ROC) curves to evaluate QTL detection performance using 100 simulation replicates for each scenario. Note that a simulation replicate is different from an experimental replicate in that it starts from a different burn-in population and has a completely different set of SNPs and QTL, therefore representing a different and independent experiment. False positive rates were defined as the percentage of neutral SNPs identified as QTL for a given signal threshold. To evaluate power (the true positive rate) for a given signal threshold, we deployed two different methods: The first method simply measures the proportion of QTL correctly identified. In simulations where effect sizes were not equal among QTL, we also used a simple variation of this method, where we weighted QTL by their effect sizes. The second method follows Kessner and Novembre (2015) and measures the proportion of genetic variance in the first generation explained by the detected QTL, thereby also taking variation in the allele frequencies into account. This method would give less weight to the detection of a low-frequency QTL compared with an intermediate-frequency QTL, because the latter would have contributed more to the initially present trait-variance in the population. Which method is more appropriate in practice depends on the specific objective of the experiment: if the goal is to identify QTL that are important to the trait regardless of their prevalence in nature, the first method should be chosen, whereas the second method should be chosen if the goal is to identify those QTL that are most important for explaining trait-variance in the initial population. We will only report the result from the first method unless the two methods generate qualitatively different results, in which case we will present both.

To produce an individual ROC curve, we first specified 100 evenly spaced signal thresholds spanning the range of observed per-SNP values of the given summary statistics (*D*, *transformed-D*, |$\underset{¯}{s}$|, |$\underset{¯}{p}$ - 0.5|) among all simulation replicates. For each threshold value, we categorized SNPs with summary statistics exceeding that threshold value as being detected as a QTL in each simulation replicate. We then took the mean power and false positive rate across all simulation replicates to add one point to the ROC curve. This process was reiterated for each threshold value.

### Data availability

All simulations were conducted within the individual-based forward genetic simulation framework SLiM 2.4.1 (Haller and Messer 2016). Our simulation pipeline can be used as a flexible tool with which readers can perform similar analysis based on parameters relevant to their study system. Two SLiM scripts were created for the simulation, one for the burn-in process and the other for the selection experiment. Two shell scripts can be used to run each of these SLiM scripts in a command-line environment, so that repeated simulations can be automated on either a local machine or a remote server. Parameter values are also defined through these shell scripts and users can easily edit them to implement custom simulation scenarios. All data analyses and visualizations are implemented using R, with packages “tidyverse” (Wickham and RStudio 2017), “data.table” (Dowle *et al.* 2019), and “cowplot” (Wilke and RStudio 2019). The SLiM and shell scripts for the simulation, along with a detailed user-guide, are available in supplementary materials and online at https://github.com/MesserLab/evolve-resequence-simulation. The data used in this paper can easily be reproduced using these scripts and the scripts can also be customized for other test cases following the user-guide. The R scripts for data analyses and visualization are also available in the same GitHub repository.

## Results

### Selection response in the standard QTL model

**Figure 1:**
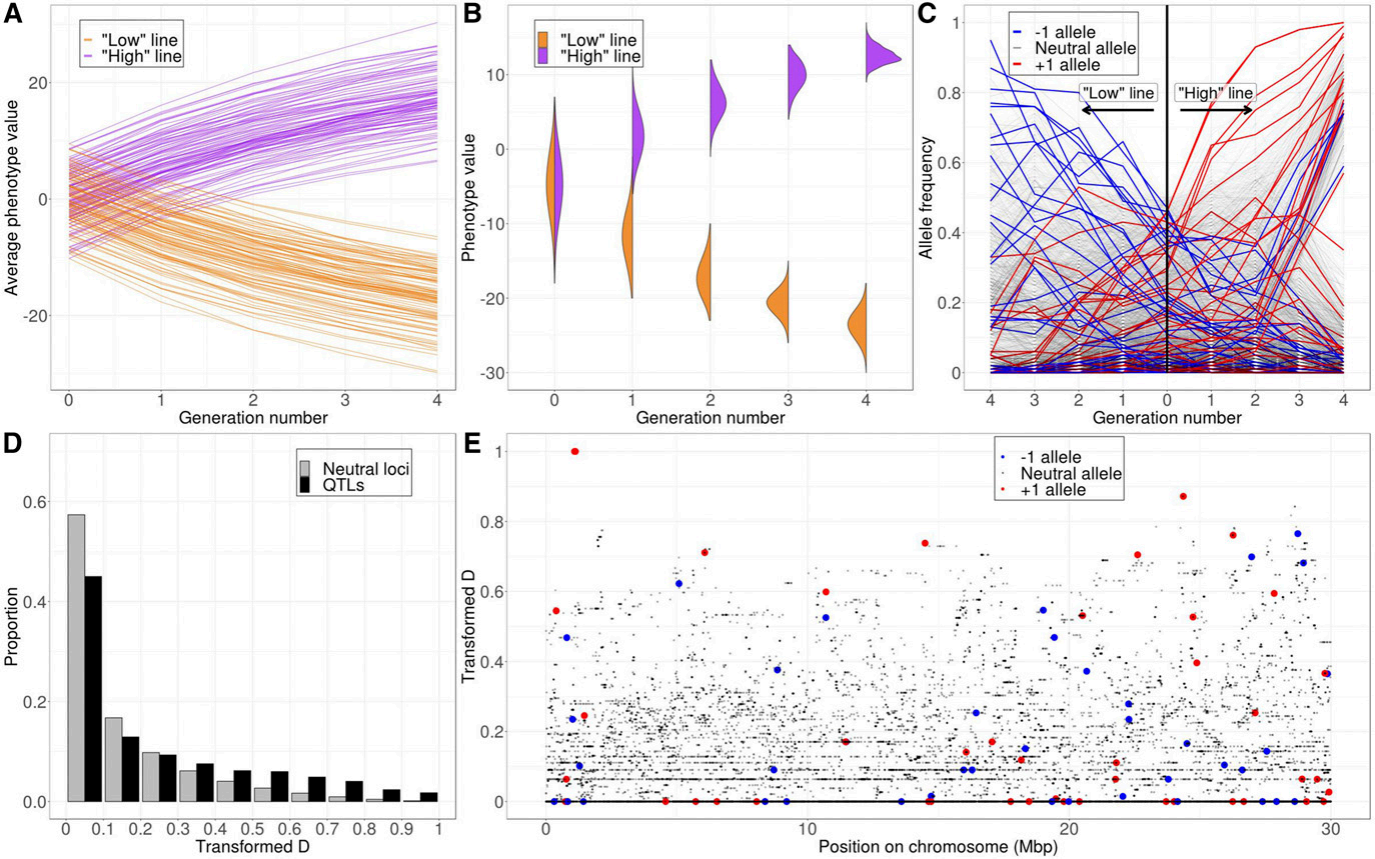
Simulation results under our standard model with 100 QTL. (A) Change in the average phenotype values in all 100 simulation replicates. Each line represents a selection line in one simulation replicate. (B) Change in the distribution of trait values in the population in one single simulation. (C) Change in sampled minor allele frequencies at neutral loci and QTL in one single simulation run. The left half of the figure shows the “low” line and the right half shows the “high” line. (D) Distribution of *transformed-D* per locus across all 100 simulation replicates grouped by neutral loci (gray bars) *vs.* QTL (black bars). (E) *transformed-D* of neutral loci and QTL along the simulated chromosome in one single simulation run.

Figure 1A shows the change in average trait value (estimated across all individuals in the population) for the “high” and “low” lines in 100 simulated experiments under our standard model with *n* = 100 QTL (Figure S2 shows results for a model with *n* = 10 QTL). Average trait values can be seen to change consistently in the selected direction, while genetic variance generally declines with diminishing returns over the course of a single experiment (Figure 1B). Note, however, that the theoretical maximum/minimum trait value in our standard QTL model would be +/−100 had all of the +1 or all of the -1 alleles, respectively, fixed in a population. The maximum trait values achieved in our simulations were typically less than a third of these maximum values. This is primarily due to the fact that many low-frequency alleles at QTL are lost due to drift or interference between neighboring QTL even when they should have been favored by selection. Overall, these results demonstrate an effective selection response on the phenotypic level in our model that is consistent among simulation replicates, suggesting that we should be able to observe meaningful evolutionary dynamics in our experimental setup.

Figure 1C shows sampled allele frequency trajectories at all SNPs in the “high” and “low” lines from a single experiment. As expected, minor alleles at the individual QTL tend to change in frequency in the selected direction (*e.g.*, in the “high” line, at those QTL where the derived allele has an effect size of +1, the derived alleles tend to increase in frequency while the ancestral alleles tend to decrease in frequency, and vice versa at those QTL where the derived allele has an effect size of -1). However, several complexities of the allele frequency dynamics are revealed in this figure: First, many alleles that should have been favored by selection do not actually rise consistently in frequency. This is particularly common for alleles that start at low frequency, which often get lost in both selection lines. But even after favored alleles reach intermediate frequency, they can still subsequently drop in frequency due to interference with other linked QTL. On the other hand, neutral alleles and alleles that are disfavored by selection can consistently rise in frequency when they are located on haplotypes with a net excess of favored alleles. These linkage effects produce dynamics that are quite different from a model of independent selective sweeps in which alleles favored by selection tend to consistently increase in frequency and neutral alleles tend to fluctuate randomly in frequency due to drift (Kessner and Novembre 2015). Also, note that under strong selection pressure, many QTL are already close to fixation after four generations and would likely fix soon within just a few more generations, so conducting the experiment for only four generations allows us to better distinguish selection from drift. Therefore, we chose to keep using four generations in our standard model, but we will explore a model with more generations in the discussion section.

Figure 1D shows the distribution of *transformed-D* across SNPs over 100 simulation runs of our standard model with *n* = 100 QTL. The distribution is heavily peaked at *transformed-D* equal or close to zero for both neutral and QTL SNPs, which is expected because the derived alleles at most SNPs will be at low frequency at the start of the experiment, and thus prone to being lost to drift in both the “high” and “low” lines. This will generally limit detection power when measured as the overall fraction of QTL identified, given that many of the initially present alleles at QTL will be lost in an experiment. However, we also see that among those SNPs with high *transformed-D* values, QTL are strongly enriched over neutral loci, suggesting that *transformed-D* should have some power in detecting QTL under this experimental setup. Figure 1E shows the distribution of *transformed-D* along the chromosome in one simulation run with 100 QTL, demonstrating that neutral SNPs with high *transformed-D* values are not necessarily always close to the QTL, but can be found across the whole chromosome.

### Performance of different detection methods under the standard QTL model

We first compared the performance of *D*, *transformed-D*, WFABC, and ApproxWF to detect the QTL in our standard model, assuming a trait comprised of 10 QTL with equal effect size. Even though SNP density is comparatively low in our standard model (∼14,000 SNPs along the 30 Mbp chromosome), the runtime of the two model-based methods still exceeded our practical limits, so we further reduced the nucleotide diversity by a factor of 10 in these simulations (for this comparison only). We then tested the model-based methods supplying them either with the entire allele frequency trajectory (*i.e.*, allele frequency estimates at all five time-points of the experiment), or only the allele frequencies at the beginning and the end of the experiment.

**Figure 2:**
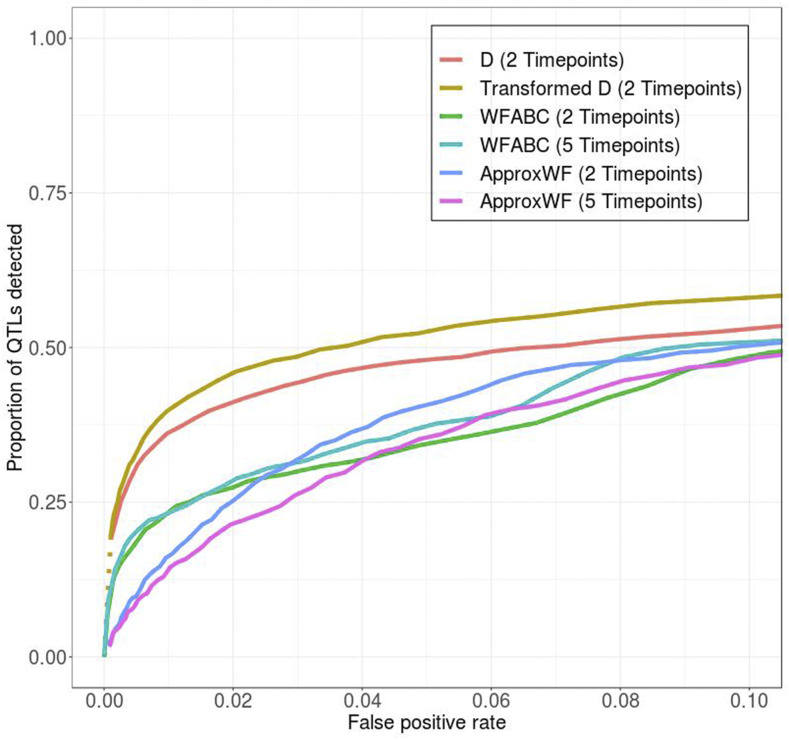
Detection power varies across detection methods used. Having the entire allele frequency trajectory slightly improves the power of WFABC but not ApproxWF, although *transformed-D* has the highest detection power regardless. This comparison used the standard model with 10 QTL and 10-times reduced nucleotide diversity due to the long run-time of the two model-based detection methods. Dotted lines: in certain simulation scenarios, multiple QTL and neutral loci get fixed in one selection line and get lost in the other. This implies that the power and false positive rate associated with the most stringent summary statistic threshold can still be quite large, and the dotted line thus represents the discontinuous transition from the origin (0,0) to the minimum non-zero power and false positive rate. Solid lines: solid lines can be interpreted as a continuous relationship between power and false positive rate, as opposed to dotted lines.

Surprisingly, WFABC and ApproxWF typically had lower power to correctly detect QTL (*i.e.*, lower true detection rate for a given false negative detection rate) than the simple *D* and *transformed-D* statistics, even when they were provided the full allele frequency trajectory at all five time points (Figure 2). This may in part be due to the fact that these methods were not explicitly developed for a divergent selection scenario resulting in two opposingly selected lines. However, when we restricted the experiment to only one direction of selection, *transformed-D* still performed better than WFABC and ApproxWF (Figure S3). One possible explanation for the poorer performance of the model-based approaches is that the allele frequency dynamics at QTL in our truncating selection scenario may not typically follow the classic sweep model these methods assume, as has already been observed in previous studies (Burke *et al.* 2010; Kessner and Novembre 2015; Franssen *et al.* 2017). Consequently, we decided to use only the *transformed-D* statistic for all further analyses in this study.

### Effects of trait architecture on detection power

#### Number of QTL affecting a trait:

**Figure 3:**
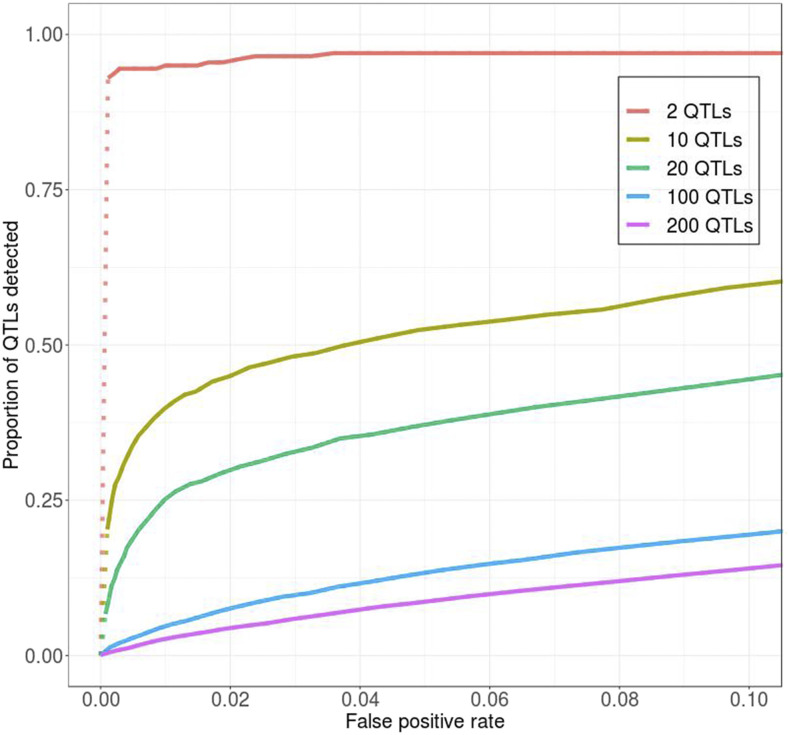
Detection power is lower when more QTL underlie the trait under selection. Solid *vs.* dotted lines: see Figure 2 caption.

To test how the number of QTL contributing to a trait affects detection power, we systematically varied the number of SNPs assigned to be QTL in our simulations. Figure 3 shows a comparison of the ROC curves among models with 2, 10, 20, 50, 100, and 200 QTL, while all other aspects of the model were kept the same as in the standard model. Consistent with previous results (Kessner and Novembre 2015), we find that larger numbers of QTL generally resulted in lower detection power, presumably due to increased interference between QTL. As the number of QTL increases, individual QTL will tend to be located closer to each other, decreasing recombination rate between them. Recombination will then be less effective at creating “optimal” haplotypes that carry a large number of favored but only few unfavored alleles. A complementary effect is that more QTL also mean less effective selection on every single one of them, because the relative contribution of each individual mutation to the overall variance in trait value gets smaller (Barton and Turelli 1989).

Note that the overall detection power is rather low in our standard model with 100 QTL. In that case, we detected only ∼13% of the QTL at a false positive rate of 0.05 (*i.e.*, 5% of the neutral loci are falsely identified as QTL). This is because most QTL start out at low frequency and have a high probability of getting lost over the course of the experiment. However, since intermediate-frequency QTL are more likely to be detected, when weighting QTL by their contribution to genetic variance in the first generation (which should be much higher for intermediate-frequency than low-frequency QTL), power improves significantly (Figure S4). For example, at the same false positive rate of 0.05, the detected QTL were responsible for more than 40% of the genetic variance present in the first generation (Figure S4).

#### QTL clustering:

In our standard model the QTL are positioned uniformly along the chromosome, as we chose them randomly from preexisting SNPs. However, for some traits, QTL could cluster along the chromosome. This is frequently observed among domestication-related traits in crops, for example (Cai and Morishima 2002; Burger *et al.* 2008). To test how such clustering affects detection power, we compared our standard QTL model with a model in which all QTL were drawn from only those SNPs that were located within a much shorter genomic sub-region 3 Mbp in length, located at the center of the chromosome.

**Figure 4:**
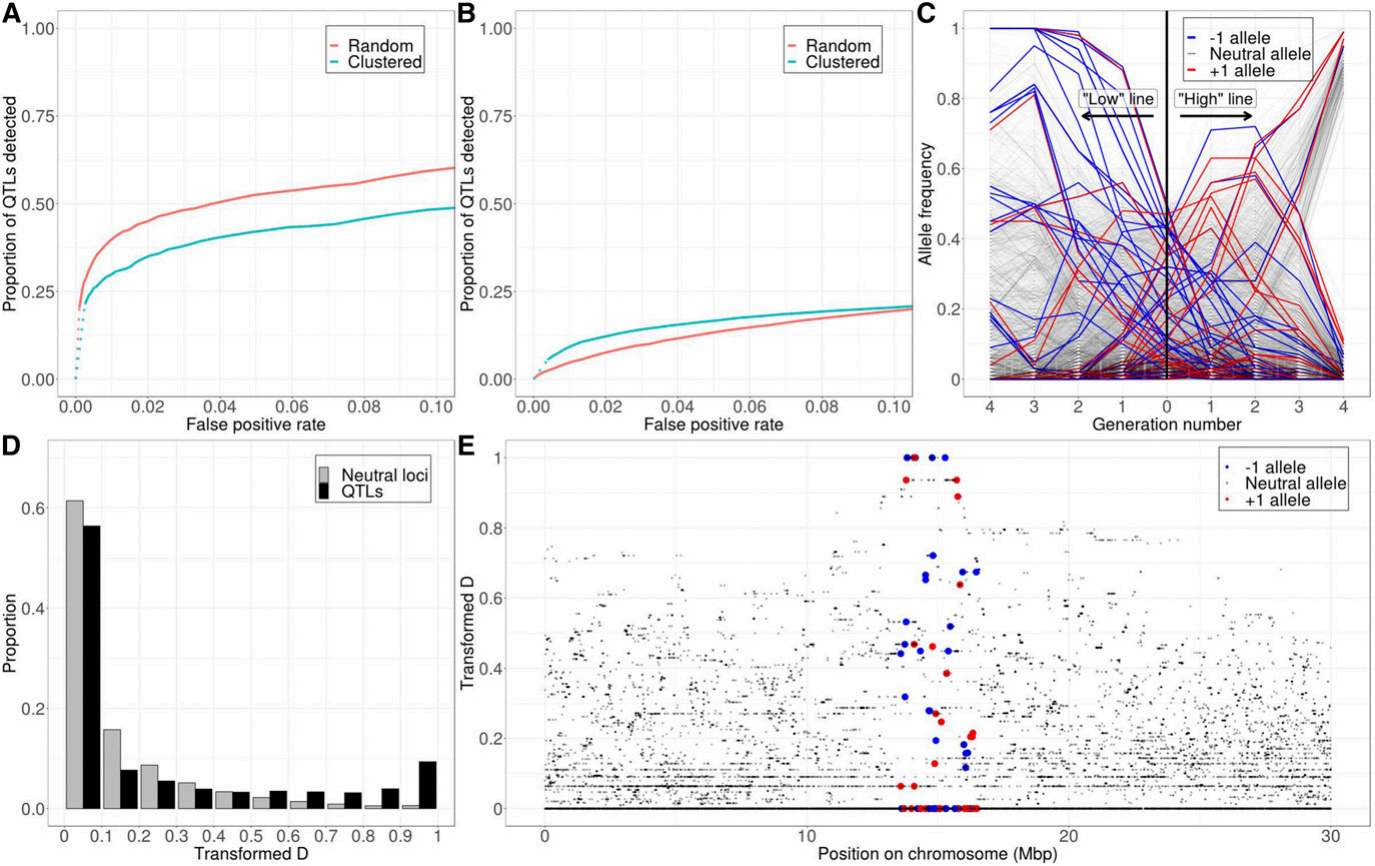
The clustering of QTL has different effects on detection power depending on the number of QTL underlying the trait under selection. (A) With 10 QTL, clustering reduces detection power. (B) With 100 QTL, clustering increases detection power except for at very high false positive rates (>0.1). (C) An example of allele frequency trajectories when 100 QTL are clustered in a small region on the chromosome. Note that many SNPs on the same haplotype quickly sweep to fixation in tandem in the “low” line, and see Figure 1C for comparison with the standard model. (D) Such haplotype sweeping results in more QTL with extreme *transformed-D* values (see Figure 1D for comparison with the standard model). (E) The distribution of *transformed-D* along the chromosome after a haplotype sweeping (see Figure 1E for comparison with the standard model). Solid *vs.* dotted lines: see Figure 2 caption.

For a model with only 10 QTL, we found that such clustering lowers detection power compared to a more uniform distribution (Figure 4A & S5A). This is likely due to increased linkage between QTL, resulting in an effectively lower rate at which recombination can create haplotypes with a large number of favored alleles. Clustering therefore has a similar effect as increasing the overall number of QTL while keeping the length of the chromosome constant.

However, this behavior becomes more complicated as the number of QTL increases further. In a model with 100 QTL, we found that the effect of clustering on detection power depends on what false positive rate is deemed tolerable. For false positive rates below 0.1, clustering actually increases detection power (Figure 4B & S5B). A possible explanation for this is that when there are many QTL clustered within a short region on the genome, it is more likely that already in the beginning of the experiment a short haplotype exists on which many alleles with effects of the same direction are co-located. Unlikely to be broken by recombination, such a haplotype will be able to quickly sweep to fixation, giving a very clear signal of being under selection (Figure 4C, 4D & 4E). Therefore, at a low false positive rate, the scenario where 100 QTL are clustered on the chromosome tends to have higher power than our standard model.

Furthermore, we note that clustering has a similar effect as decreasing the recombination rate in our simulation. Thus, our results contradict that of Kessner and Novembre (2015), who concluded that increasing recombination always increases detection power. However, our results are consistent with a rich body of literature which shows that increased recombination does not always lead to higher rate of adaptation because it can also destroy “good” haplotypes that are initially present (Slatkin 1975; Charlesworth and Charlesworth 1979; Kirkpatrick and Barton 2006).

#### Effect size distribution:

Our standard model assumes equal effect sizes of derived alleles of either +1 or -1 at all individual QTL. We chose this simplistic model because little is known about the actual effect size distributions of complex traits in most biological systems and thus there is not a single ideal distribution. Also, results on other aspects of the trait architecture will be more difficult to interpret if we add an additional layer of stochasticity to the simulations by randomly assigning uneven effect sizes. In reality, however, effect sizes will typically vary among QTL, and one commonly used model for this is an exponential distribution for effect sizes (Orr 1998; Otto and Jones 2000).

**Figure 5:**
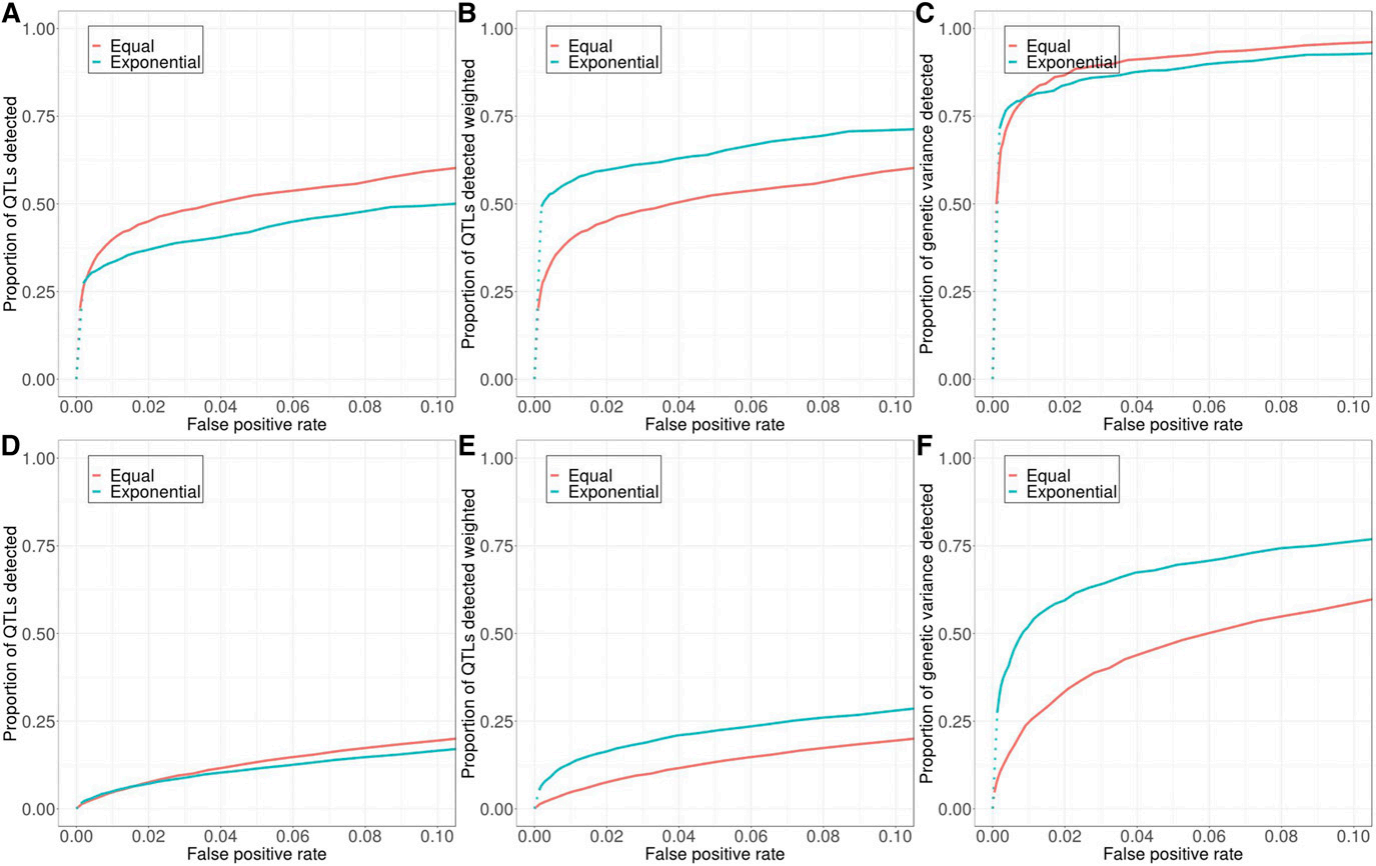
An exponential distribution of effect size affects detection power differently depending on how power is evaluated and how many QTL underlie the selected trait. All comparisons are conducted with the standard model of equal effect sizes. Top: 10 QTL. Bottom: 100 QTL. (A) (D) Exponential distribution of effect size decreases power when measured by the proportion of QTL detected. (B) (E) By contrast, exponential distribution of effect size increases power when measured by the proportion of QTL detected, weighted by their effect sizes. (C) (F) When power is measured by the proportion of genetic variance explained, an exponential distribution of effect size yields similar power when there are 10 QTL but increases power when there are 100 QTL. Solid *vs.* dotted lines: see Figure 2 caption.

Figure 5 shows how QTL detection power is affected when effect sizes in our standard model are no longer assigned equal values, but instead are drawn from an exponential distribution with means +1 or -1, respectively. Whether this increases or decreases detection power depends on how we define our measure of power. If power is defined simply as the proportion of QTL detected regardless of their effect sizes, power is lower in the model with exponentially distributed effect sizes compared with our standard model assuming constant effects (Figure 5A & 5D). One possible explanation for this is that in the exponentially distributed model, there will be many QTL with small effects that are practically neutral and are likely to get lost due to drift or interference. This means that there would be a smaller proportion of “effective” QTL that can still be detected overall.

However, when we weight individual QTL by their effect sizes, these small-effect QTL will contribute minimally to power, whereas the few large-effect QTL become much more important (Chevalet 1994). Similar to the effect of lowering the number of QTL as discussed above, these few large-effect QTL will interfere less with each other because they are less densely distributed on the chromosome and are more likely to be detected. As a result, when QTL are weighted by their effect sizes, the model with exponentially distributed effect sizes yields higher power than the constant effect size model (Figure 5B & 5E).

When we measure the proportion of genetic variance explained by detected QTL in the first generation, exponentially distributed effect sizes have two opposing effects. On the one hand, as discussed above, large-effect QTL are more likely to be detected, and they contribute more to the genetic variance given the same starting frequency. On the other hand, large-effect QTL are less likely to get lost due to drift or interference even when they start at low frequency, so intermediate-frequency QTL are less overrepresented among detected QTL (Figure S6), which would then decrease the proportion of initial genetic variance explained by the detected QTL compared to our standard model. When there are fewer QTL, the first effect is weaker than the second (Figure S6A & S6B), so detection power becomes similar between the standard and exponential models (Figure 5C). When there are more QTL, there is also more interference among large-effect QTL and thus the second effect becomes weaker (Figure S6C & S6D), resulting in higher power for the exponential model (Figure 5F).

#### Allele frequency distribution:

In our standard QTL model, we have assumed that the allele frequency distribution at QTL resemble those of neutral SNPs. However, prior selection on the trait could drastically shift this distribution from the neutral expectation. Although selection is ubiquitous in most traits of interest in natural populations, we choose not to model selection explicitly in our burn-in stage because **a)** we still know very little about what an appropriate assumption on selection in natural populations would be, and **b)** selection can significantly alter the starting population in many aspects, including the allele frequency distribution, linkage disequilibrium, and effect size distribution, so it would be difficult to isolate the effect of a single factor on the detection power.

**Figure 6:**
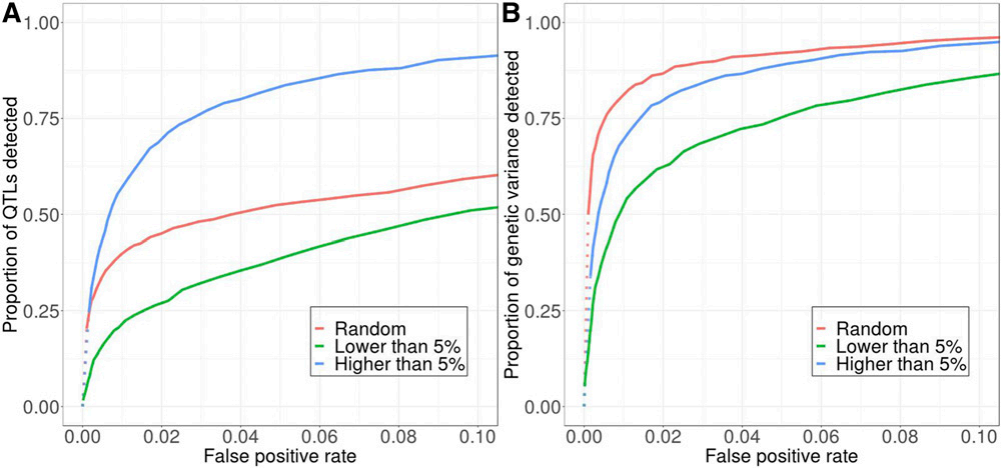
Starting frequency of minor alleles affects power differently depending on how power is evaluated. (A) Power evaluated as proportion of QTL detected. (B) Power evaluated as proportion of genetic variance explained by detected QTL. Models with 10 QTL are used in this figure. Solid *vs.* dotted lines: see Figure 2 caption.

Nevertheless, we note that theory predicts that certain selection scenarios tend to drive allele frequencies toward certain directions. For example, long-term stabilizing selection is likely to keep allele frequencies at QTL at low frequencies, whereas balancing selection tends to drive them toward intermediate frequencies. Therefore, we decide to study two rather extreme cases of allele frequency distribution as an attempt to qualitatively assess how non-neutral processes prior to the selection experiment can affect QTL detection power through shifting allele frequency distribution. Namely, we only selected SNPs with minor allele frequencies above or below a certain cutoff to become QTL in the simulation. First, we restricted our selection to SNPs with minor allele frequency lower than 5%. In this case, detection power decreased consistently regardless of the method to evaluate power (Figure 6). This is presumably because these alleles are more likely to get lost due to drift or interference regardless of the direction of selection.

Conversely, when we restricted our selection to only those SNPs with minor allele frequency higher than 5%, these minor alleles at QTL are less likely to get lost. When they are favored by selection, they are also more likely to be recombined together with other favored alleles. This is consistent with the observation that a higher proportion of total QTL can be detected in such a scenario (Figure 6A). However, the result is different when we evaluate power by the proportion of the starting genetic variance (Figure 6B). The reason is that in the standard model, QTL that start at intermediate frequencies are more likely to be detected and their detection can explain a higher proportion of starting genetic variance (Figure S7A). When all QTL start at more intermediate frequencies, they all explain a similar proportion of the starting genetic variance, and the likelihood of them being detected becomes more independent of their starting frequencies (Figure S7B), so even though more QTL can be detected, the proportion of initial genetic variance explained ends up lower.

#### Dominance:

Previous QTL models have typically assumed additive effects between the two alleles at individual QTL, which is what we also adopted in our standard model. However, dominance effects at QTL, as well epistatic effects among QTL, could play an important role in many traits (Shao *et al.* 2008; Mackay 2014; Chen *et al.* 2015).

**Figure 7:**
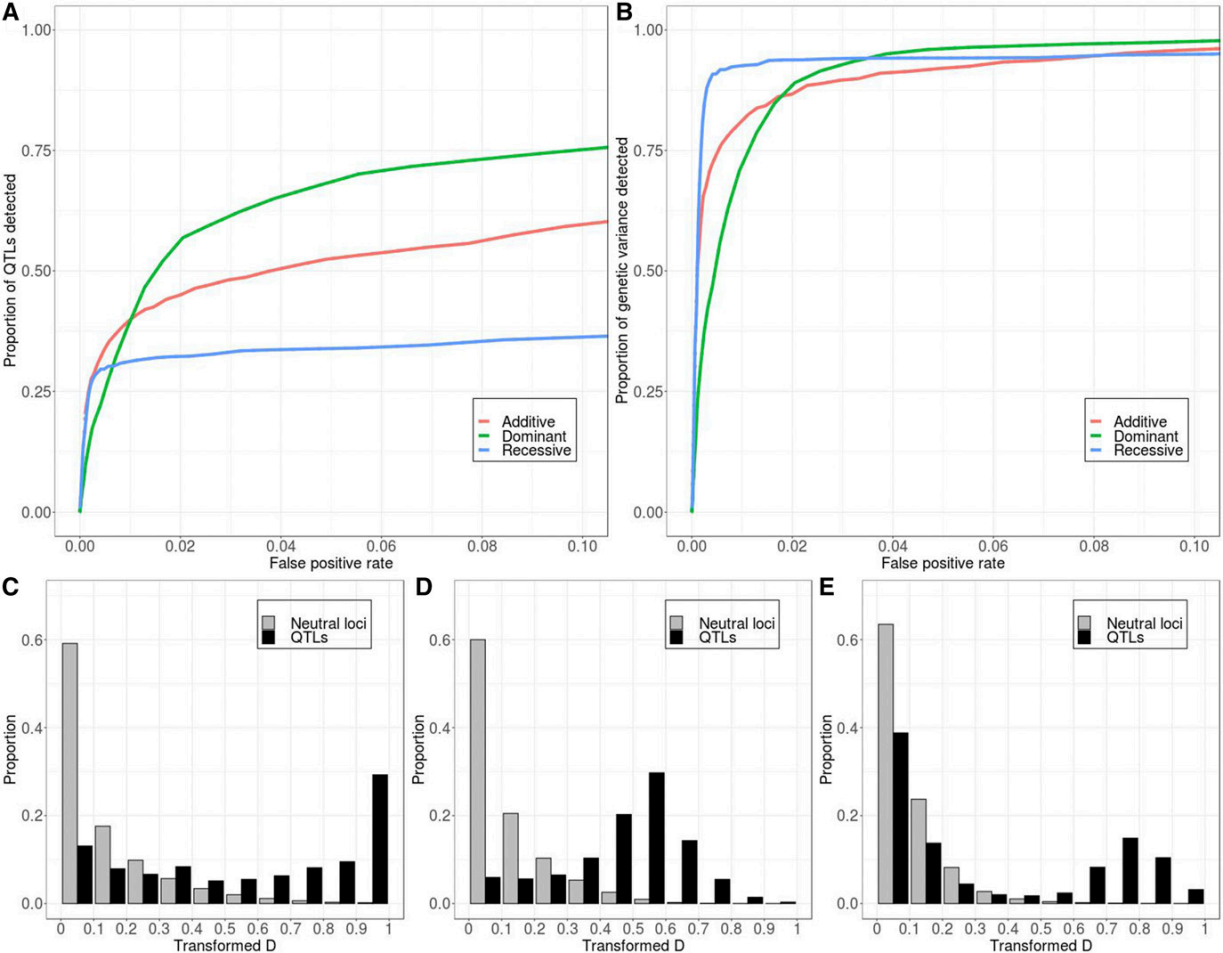
Dominance affects power differently depending on how power is evaluated and what level of false positive rate is tolerated. Top: ROC curves (A) Power evaluated as proportion of QTL detected. (B) Power evaluated as proportion of genetic variance explained by detected QTL. Bottom: Distribution of *transformed-D* over 100 simulation replicates (C) Additive. (D) Dominant. (E) Recessive. Models with 10 QTL are used in this figure. Additive: heterozygotes express intermediate phenotype. Dominant: heterozygotes express the same phenotype as derived homozygotes. Recessive: heterozygotes express the same phenotype as ancestral homozygotes. Solid *vs.* dotted lines: see Figure 2 caption.

We first tested how dominance relationships affect QTL detection power in our model. Given how our QTL are initially assigned, derived alleles tend to be the minor alleles in our model. When these derived alleles are completely dominant, heterozygotes will exhibit the same phenotype as derived homozygotes, and selection thus cannot distinguish between them. As a result, positively selected derived alleles tend to first increase in frequency, but then accumulate at intermediate frequencies. This produces a less conspicuous signal of selection than for alleles that reach higher frequencies over the course of the experiment (Figure 7C & 7D). At low false positive rates, detection power is therefore lower in this scenario than in the standard scenario where alleles are assumed to be additive (Figure 7A & 7B). At a higher false positive rate, however, a crossover pattern in the power curves is observed (Figure 7A & 7B), since lower frequency alleles are less likely to be lost due to interference than in the standard model.

When derived alleles are completely recessive, only those with high starting frequency can form homozygotes and thus be visible to selection (Figure S8). All other derived alleles behave essentially like neutral alleles initially (Figure 7C & 7E). As a result, an overall lower number of QTL can be detected (Figure 7A). However, these high-frequency QTL are also the ones that contribute most to genetic variance in the first generation. Therefore, the proportion of genetic variance detected is even higher than in the standard model (Figure 7B). These results demonstrate again how a relatively simple aspect of the trait architecture - here the dominance relationship at individual QTL - can affect detection power in complex ways, where the direction of the effect depends on the false positive rate and the definition being used to measure power.

#### Epistasis:

We next tested how epistatic interaction among QTL can affect detection power. For simplicity, we only considered pairwise epistasis here. We further restricted our analyses to additive-by-additive epistasis (*i.e.*, the effects of the two alleles at an individual QTL are always additive if genotypes at other QTL are fixed). This helps us avoid the potentially confounding effect of dominance. Under these assumptions, we tested the effect of epistasis with our 10 QTL model, where we randomly selected five epistatic QTL-pairs in each simulation. We explored separately the effect of four major types of epistasis, including synergistic, antagonistic, sign, and reciprocal sign epistasis. Within each type, we further created a “weak” and a “strong” scenario, based on the level of deviation from the non-epistatic model (Table S3). To evaluate power for a given epistasis scenario, we simply measured the overall proportion of the 10 QTL we detected, since the effect of each individual QTL is difficult to quantify.

**Figure 8:**
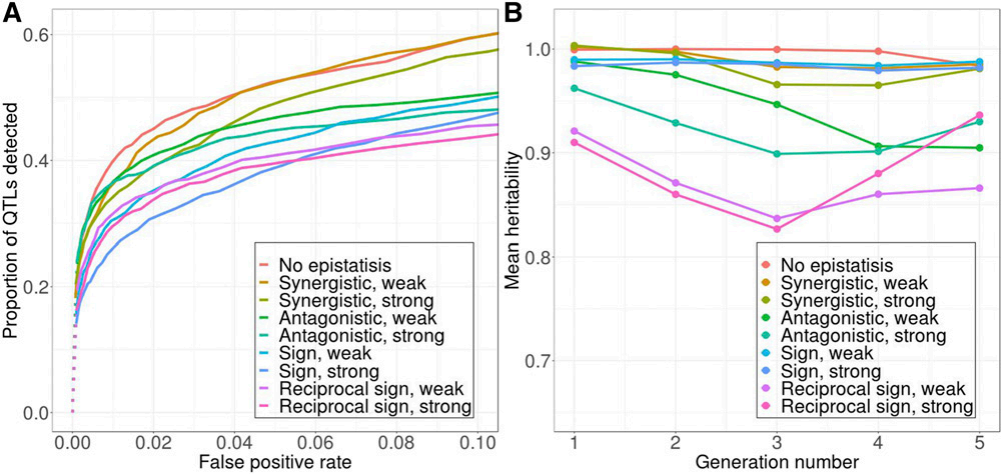
(A) Pairwise epistasis always decreases QTL detection power, but this effect is a lot stronger in certain epistatic scenarios than others. (B) Estimates of narrow-sense heritability for a given epistasis scenario at a given time-point are calculated from the breeder’s equation (Lush 1943), averaged across all 100 simulation replicates. Solid *vs.* dotted lines: see Figure 2 caption.

Our simulations show that pairwise epistasis generally decreases the power in QTL detection, as would be expected (Figure 8A). However, a lot of variability exists among different epistasis scenarios. Within each type of epistasis, the more it deviates from the non-epistatic case, the less detection power is generally attained. Among different types of epistasis, synergistic epistasis tends to have higher power, while sign and reciprocal sign epistasis tend to have lower power. Since a major effect of epistasis is introducing epistatic genetic variance and lowering the trait’s narrow-sense heritability (*h*^2^), we examined how narrow-sense heritability varied across our epistasis scenarios, and found that narrow-sense heritability is a good predictor of power (Figure 8B). Scenarios that create lower narrow-sense heritability (or higher epistatic genetic variance) tend to have lower QTL detection power. However, sign epistasis is an exception to this rule. It has relatively high narrow-sense heritability throughout the experiment, but its detection power remained low in our simulations.

## Discussion

In this paper, we constructed an explicit QTL model using a forward simulation framework to study the power to detect QTL in a short-term E&R experiment with intense truncating selection. Although in many ways our model is highly idealized, it allowed us to qualitatively assess how different fundamental aspects of trait architecture can affect the expected power of such experiments. This relationship is complex and often unintuitive, and we demonstrated that few results hold universally, except perhaps for the fact that as the number of QTL affecting the trait increases, the power to detect them always decreases (Figure 3). Other results are often conditional on the false positive rate, the precise way in which power is measured, and various aspects of trait architecture. For example, we found that the clustering of QTL along a chromosome decreases detection power when there are fewer QTL, but increases power at low false positive rates when there are more QTL (Figure 4). An exponential distribution in effect size lowers the proportion of QTL detected compared to when effect sizes are equal at all QTL, but increases power when QTL are weighted by their effect sizes (*i.e.*, we have a better chance at detecting a substantial portion of the genetic basis of a trait, Figure 5). When minor alleles at QTL are skewed toward lower frequencies, detection power is generally decreased, but when they start at higher frequencies, detection power is increased only when it is measured by the proportion of QTL detected (Figure 6). When derived alleles are dominant, detection power is increased at higher false positive rates but decreased at lower (Figure 7). When they are recessive, detection power is decreased only when it is evaluated by the proportion of QTL detected (Figure 7). Epistasis tends to always decrease power, although a lot of variation exists among different types of epistasis (Figure 8). In general, we found that except for the rare cases where the trait in question has a very simple genetic architecture, short E&R experiments with intense truncating selection spanning only four generations tend to have low power to detect the full set of QTL present in the starting population and suffer from high false positive rates. However, while such a setup is clearly not ideal for characterizing the full genomic architecture of traits, sequencing the preserved specimens in an experiment that has already been conducted for other purposes can still be useful in detecting the QTL that contribute substantially to the initial trait variance in the experimental population.

Our results highlight the complex nature of the mechanisms involved that determine the detection power of E&R experiments and thus invoke caution when interpreting the results of such experiments. Particularly, we demonstrate that certain QTL and certain trait architectures are more likely to be discovered through E&R experiments than others. Therefore, making conclusions on the trait architecture solely based on the detected QTL from an E&R experiment can be misleading. In addition, simple simulations that do not take many aspects of trait architecture into account are often used for the estimation of false positive rate in E&R experiments. Since trait architecture plays an essential role in determining the relationship between power and false positive rate, we argue that such an approach can lead to largely inaccurate estimates.

From the perspective of researchers who plan to perform E&R studies, a key question might be which experimental designs could optimize detection power (Kofler and Schlötterer 2014; Baldwin-Brown *et al.* 2014; Kessner and Novembre 2015). Our results suggest that there is not a single optimal value for each experimental design parameter. Instead, what the best strategy is can depend critically on the genetic architecture of the trait and other experimental design parameters. For example, we tested how much our simulated experiment can benefit from adding replicated populations and more generations of selection in our standard model with 10 and 100 QTL. We found that having two or five replicated populations alone does little in improving the detection power (Figure S9A & S9C), since allele frequencies at both QTL and neutral loci change rather deterministically (*i.e.*, it is always the same loci that experience high frequency change) due to the strong selection in our experiment. Consequently, different replicates often end up showing the same results (Figure S10). Extending the experiment for five more generations alone can improve power at higher false positive rate but reduces power at low false positive rate (Figure S9B & S9D), because many loci, QTL and neutral ones alike, go to fixation in one selection line and get lost in the other (Figure S10). A combination of replication and extension of the experiment, however, can significantly improve detection power at low false positive rate (Figure S9B & S9D), because after many QTL go to fixation, dynamics in neutral allele frequencies become more stochastic and different replicates start to provide information that is complementary to each other (Figure S10). This effect is particularly prominent when there are fewer QTL (Figure S9B, Figure S10A). We do note here that caution should be taken when interpreting our results with regard to experimental replications. Since all of our experimental replicates start from the same population at the first generation and the broad-sense heritability of the trait is one, stochasticity in allele frequency change is generally low (Figure S10). In reality, differences in initial allele frequencies and environmental effects are likely to introduce more stochasticity to allele frequency trajectories, and therefore replication is expected to improve power more significantly than in our simulations. In addition, we simply took the average value of *transformed-D* across experimental replicates, so other QTL detection methods specifically taking experimental replications into account (*e.g.*, Kelly and Hughes 2019) may lead to further gain in power.

**Figure 9:**
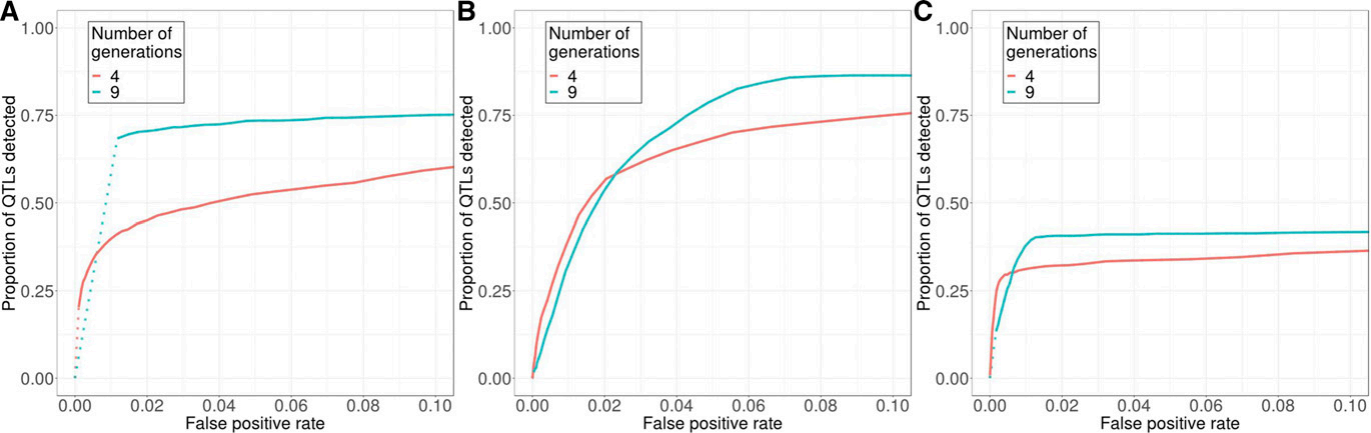
The choice of optimal experimental design depends on the underlying trait architecture. Extending the selection experiment from four to nine generations substantially increases detection power at higher false positive rates when the effect of derived allele is (A) additive, but such effect is minimal when the derived allele is either (B) completely dominant or (C) completely recessive. Models with 10 QTL are used. Additive: heterozygotes express intermediate phenotype. Dominant: heterozygotes express the same phenotype as derived homozygotes. Recessive: heterozygotes express the same phenotype as ancestral homozygotes. Solid *vs.* dotted lines: see Figure 2 caption.

In another example, running the selection experiment for an additional five generations can significantly improve detection power at higher false positive rate in our standard model with 10 QTL, since many QTL are still segregating at intermediate frequency after just four generations of selection. However, the gain in power will be minimal if derived alleles are either dominant or recessive, because during the additional generations, selection would become less effective in the dominant case, while most of the genetic variance will already have been depleted in the recessive case (Figure 9).

Since the parameter space of our model is vast, the idealized scenarios presented in this paper can only cover a tiny proportion of what is actually possible. To allow researchers to study alternative models, we provide a flexible simulation framework that is highly customizable with regard to trait architecture, experimental design, and parameters of the genomic background (described in the Supplementary Materials). When some information on the expected trait architecture is available, different experimental designs can be simulated to find the optimal setup for the given architecture. Without such *a priori* information, a range of architectures may need to be simulated to obtain a general sense of the range of power that could be achieved and what types of architectures are likely to be detectable under different experimental designs.

While several specialized tools have already been designed for such applications (Neuenschwander *et al.* 2008; Zanini and Neher 2012; Kofler and Schlötterer 2014; Kessner and Novembre 2015; Vlachos and Kofler 2018), we believe that our approach provides key advantages by implementing its simulations in the flexible SLiM framework (Haller and Messer 2016, 2019), one of the most widely used and well-tested frameworks for forward genetic simulation to date. SLiM simulations are fully scriptable, allowing the user to model a wide range of evolutionary scenarios that can include high levels of genetic and ecological realism, while the underlying simulation engine has been highly optimized over the years. All SLiM configuration scripts developed in this study are provided in the Supplementary Materials, together with a comprehensive user-guide explaining how these scripts may be adjusted for custom scenarios.

One perhaps surprising result of our analyses was that a simple summary statistic, *transformed-D*, had better performance than two of the more sophisticated, model-based methods explicitly devised for detecting positive selection from time-series data. This is presumably because these methods were built on the assumption that selection produces independent selective sweeps at individual QTL. However, when selection operates on a polygenic trait, allele frequency trajectories at its QTL will often be quite distinct from those expected in a model of individual sweeps with fixed selection coefficients (Burke *et al.* 2010; Kessner and Novembre 2015; Franssen *et al.* 2017). In addition, QTL are usually not freely recombining, further complicating allele frequency trajectories due to interference effects. Therefore, simple summary statistics that do not rely on potentially inaccurate assumptions about the detailed form of the temporal allele frequency trajectories may actually work better in these scenarios than some of these more sophisticated model-based methods. We note that time-series data indeed contain valuable additional information that is not available from the simple *transformed-D* statistic (Franssen *et al.* 2017), and that some other model-based methods may be able to capitalize from such information (*e.g.*, Iranmehr *et al.* 2017; Buffalo and Coop 2019), but it is not our goal in this paper to exhaustively test all of these methods.

The low detection power in our study partly reflects the limitations common to most E&R studies, namely the high genetic drift caused by small population sizes and the polygenic nature of most traits of interest (Long *et al.* 2015). Compared with earlier power analyses of E&R experiments, however, detection power was generally lower in our selection model. This can be attributed to the following factors. First, selection is quite extreme in our model, while the length of the experiment is very short. Such an experimental design would likely be considered as ineffective when working with an insect species such as flies, but may be the only realistic choice for larger organisms with longer generation times. Second, our standard model does not include replicated experiments, which can significantly improve the power when combined with a longer experiment (Figure S9). But again, this may be infeasible for larger and longer-living species. Third, we assumed a higher level of linkage disequilibrium and thus stronger interference effects than previous studies that focused on insect systems. We also note that for simplicity we set the broad-sense heritability to a value of one in our model, which should generally lead to an overestimation of power. This can easily be modified in our SLiM simulations if a more realistic estimation of power for a specific organism and trait of interest is desired.

## Conclusion

Overall, we find that although short E&R experiments with strong truncating selection have low power in general, they can still provide some utility in identifying at least part of the genetic basis of the selected trait, especially if the goal is to detect those QTL that contribute most to the observed trait variance in the population (*i.e.*, large effect QTL present at high population frequency). However, we have also shown that detection power can vary substantially with the genetic architecture of the trait. This presents a problem of circular reasoning, because the architecture of the trait will likely be unknown prior to the experiment yet the power to identify its genetic basis should be biased against certain classes of QTL (*e.g.*, those that are dominant/recessive, have epistatic interactions, or are present at low initial frequencies). Thus, caution is warranted when trying to make general conclusions about the architecture of the selected trait, based solely on the subset of QTL that were identified in an E&R experiment. Future studies will hopefully improve our understanding of what types of trait architectures are more prevalent in nature and thereby help us build better priors for the interpretation of E&R experiments.

## References

[bib1] Backström N., Forstmeier W., Schielzeth H., Mellenius H., Nam K., 2010 The recombination landscape of the zebra finch *Taeniopygia guttata* genome. Genome Res. 20: 485–495. 10.1101/gr.101410.109 20357052 PMC2847751

[bib2] Baldwin-Brown J. G., Long A. D., and Thornton K. R., 2014 The power to detect quantitative trait loci using resequenced, experimentally evolved populations of diploid, sexual organisms. Mol. Biol. Evol. 31: 1040–1055. 10.1093/molbev/msu048 24441104 PMC3969567

[bib3] Barrett R. D. H., Paccard A., Healy T. M., Bergek S., Schulte P. M., 2011 Rapid evolution of cold tolerance in stickleback. Proc. Biol. Sci. 278: 233–238. 10.1098/rspb.2010.0923 20685715 PMC3013383

[bib4] Barrett R. D. H., Laurent S., Mallarino R., Pfeifer S. P., Xu C. C. Y., 2019 Linking a mutation to survival in wild mice. Science 363: 499–504. 10.1126/science.aav3824 30705186

[bib5] Barrick J. E., Yu D. S., Yoon S. H., Jeong H., Oh T. K., 2009 Genome evolution and adaptation in a long-term experiment with *Escherichia coli*. Nature 461: 1243–1247. 10.1038/nature08480 19838166

[bib6] Barton N. H., and Turelli M., 1989 Evolutionary quantitative genetics: How little do we know? Annu. Rev. Genet. 23: 337–370. 10.1146/annurev.ge.23.120189.002005 2694935

[bib7] Buffalo V., and Coop G., 2019 The Linked Selection Signature of Rapid Adaptation in Temporal Genomic Data. Genetics 213: 1007–1045. 10.1534/genetics.119.302581 31558582 PMC6827383

[bib8] Burger J. C., Chapman M. A., and Burke J. M., 2008 Molecular insights into the evolution of crop plants. Am. J. Bot. 95: 113–122. 10.3732/ajb.95.2.113 21632337

[bib9] Burke M. K., Dunham J. P., Shahrestani P., Thornton K. R., Rose M. R., 2010 Genome-wide analysis of a long-term evolution experiment with *Drosophila*. Nature 467: 587–590. 10.1038/nature09352 20844486

[bib10] Cai H., and Morishima H., 2002 QTL clusters reflect character associations in wild and cultivated rice. Theor. Appl. Genet. 104: 1217–1228. 10.1007/s00122-001-0819-7 12582574

[bib11] Castro J. P. L., Yancoskie M. N., Marchini M., Belohlavy S., Kučka M., 2019 An integrative genomic analysis of the Longshanks selection experiment for longer limbs in mice. Elife 8: e42014 31169497 10.7554/eLife.42014 PMC6606024

[bib12] Chan Y. F., Jones F. C., McConnell E., Bryk J., Bünger L., 2012 Parallel selection mapping using artificially selected mice reveals body weight control loci. Curr. Biol. 22: 794–800. 10.1016/j.cub.2012.03.011 22445301

[bib13] Charlesworth D., and Charlesworth B., 1979 Selection on recombination in clines. Genetics 91: 581–589. 17248899 10.1093/genetics/91.3.581 PMC1216851

[bib14] Chen X., Kuja-Halkola R., Rahman I., Arpegård J., Viktorin A., 2015 Dominant genetic variation and missing heritability for human complex traits: Insights from twin *vs.* genome-wide common SNP models. Am. J. Hum. Genet. 97: 708–714. 10.1016/j.ajhg.2015.10.004 26544805 PMC4667127

[bib15] Chevalet C., 1994 An approximate theory of selection assuming a finite number of quantitative trait loci. Genet. Sel. Evol. GSE 26: 379–400. 10.1186/1297-9686-26-5-379

[bib16] Christie M. R., Marine M. L., Fox S. E., French R. A., and Blouin M. S., 2016 A single generation of domestication heritably alters the expression of hundreds of genes. Nat. Commun. 7: 10676 10.1038/ncomms10676 26883375 PMC4757788

[bib17] Coltman D. W., O’Donoghue P., Jorgenson J. T., Hogg J. T., Strobeck C., 2003 Undesirable evolutionary consequences of trophy hunting. Nature 426: 655–658. 10.1038/nature02177 14668862

[bib18] Conover D. O., and Munch S. B., 2002 Sustaining fisheries yields over evolutionary time scales. Science 297: 94–96. 10.1126/science.1074085 12098697

[bib20] Dowle M., A. Srinivasan, J. Gorecki, M. Chirico, P. Stetsenko, *et al.*, 2019 data.table: Extension of “data.frame.”

[bib21] Fallahsharoudi A., de Kock N., Johnsson M., Bektic L., Ubhayasekera S. J. K. A., 2017 Genetic and targeted eQTL mapping reveals strong candidate genes modulating the stress response during chicken domestication. G3 (Bethesda). 7: 497–504. 10.1534/g3.116.037721 27974436 PMC5295596

[bib22] Ferrer-Admetlla A., Leuenberger C., Jensen J. D., and Wegmann D., 2016 An approximate Markov model for the Wright-Fisher diffusion and its application to time series data. Genetics 203: 831–846. 10.1534/genetics.115.184598 27038112 PMC4896197

[bib23] Fisher R. A., and Ford E. B., 1947 The spread of a gene in natural conditions in a colony of the moth Panaxia dominula L. Heredity 1: 143–174. 10.1038/hdy.1947.11

[bib24] Foll M., Shim H., and Jensen J. D., 2015 WFABC: a Wright–Fisher ABC-based approach for inferring effective population sizes and selection coefficients from time-sampled data. Mol. Ecol. Resour. 15: 87–98. 10.1111/1755-0998.12280 24834845

[bib25] Franssen S. U., Kofler R., and Schlötterer C., 2017 Uncovering the genetic signature of quantitative trait evolution with replicated time series data. Heredity 118: 42–51. 10.1038/hdy.2016.98 27848948 PMC5176121

[bib26] Fuller R. C., Baer C. F., and Travis J., 2005 How and When Selection Experiments Might Actually be Useful. Integr. Comp. Biol. 45: 391–404. 10.1093/icb/45.3.391 21676785

[bib27] Garland T., and Rose M. R., 2009 *Experimental Evolution: Concepts*, *Methods*, *and Applications of Selection Experiments*. University of California Press, Berkeley, CA.

[bib28] Gibson G., 2012 Rare and common variants: twenty arguments. Nat. Rev. Genet. 13: 135–145. 10.1038/nrg3118 22251874 PMC4408201

[bib29] Gutierrez A. P., Yáñez J. M., and Davidson W. S., 2016 Evidence of recent signatures of selection during domestication in an Atlantic salmon population. Mar. Genomics 26: 41–50. 10.1016/j.margen.2015.12.007 26723557

[bib30] Haller B. C., and Messer P. W., 2016 SLiM 2: Flexible, interactive forward genetic simulations. Mol. Biol. Evol. 34: 230–240. 10.1093/molbev/msw211 27702775

[bib32] Haller B. C., and Messer P. W., 2019 SLiM 3: Forward genetic simulations beyond the Wright-Fisher model. Mol. Biol. Evol. 36: 632–637. 10.1093/molbev/msy228 30517680 PMC6389312

[bib33] Hansen T. F., 2006 The evolution of genetic architecture. Annu. Rev. Ecol. Evol. Syst. 37: 123–157. 10.1146/annurev.ecolsys.37.091305.110224

[bib34] Hill W. G., and Robertson A., 1966 The effect of linkage on limits to artificial selection. Genet. Res. 8: 269–294. 10.1017/S0016672300010156 5980116

[bib35] Hill W. G., and Caballero A., 1992 Artificial selection experiments. Annu. Rev. Ecol. Syst. 23: 287–310. 10.1146/annurev.es.23.110192.001443

[bib36] Houde A. E., 1994 Effect of artificial selection on male colour patterns on mating preference of female guppies. Proc. Biol. Sci. 256: 125–130. 10.1098/rspb.1994.0059

[bib37] Iranmehr A., Akbari A., Schlötterer C., and Bafna V., 2017 Clear: Composition of Likelihoods for Evolve and Resequence Experiments. Genetics 206: 1011–1023. 10.1534/genetics.116.197566 28396506 PMC5499160

[bib38] Johansson A. M., Pettersson M. E., Siegel P. B., and Carlborg Ö., 2010 Genome-wide effects of long-term divergent selection. PLoS Genet. 6: e1001188 10.1371/journal.pgen.1001188 21079680 PMC2973821

[bib39] Kelly J. K., and Hughes K. A., 2019 Pervasive linked selection and intermediate-frequency alleles are implicated in an evolve-and-resequencing experiment of *Drosophila simulans*. Genetics 211: 943–961. 10.1534/genetics.118.301824 30593495 PMC6404262

[bib40] Kessner D., and Novembre J., 2015 Power analysis of artificial selection experiments using efficient whole genome simulation of quantitative traits. Genetics 199: 991–1005. 10.1534/genetics.115.175075 25672748 PMC4391575

[bib41] Kirkpatrick M., and Barton N., 2006 Chromosome inversions, local adaptation and speciation. Genetics 173: 419–434. 10.1534/genetics.105.047985 16204214 PMC1461441

[bib42] Kofler R., and Schlötterer C., 2014 A guide for the design of evolve and resequencing studies. Mol. Biol. Evol. 31: 474–483. 10.1093/molbev/mst221 24214537 PMC3907048

[bib43] Lang G. I., Rice D. P., Hickman M. J., Sodergren E., Weinstock G. M., 2013 Pervasive genetic hitchhiking and clonal interference in forty evolving yeast populations. Nature 500: 571–574. 10.1038/nature12344 23873039 PMC3758440

[bib44] Long A., Liti G., Luptak A., and Tenaillon O., 2015 Elucidating the molecular architecture of adaptation via evolve and resequence experiments. Nat. Rev. Genet. 16: 567–582. 10.1038/nrg3937 26347030 PMC4733663

[bib80] Lush J. L., 1943 *Animal Breeding Plans*, The Lowa State College Press, Ames, IA.

[bib45] Mackay T. F., 2009 Q&A: Genetic analysis of quantitative traits. J. Biol. 8: 23 10.1186/jbiol133 19435484 PMC2689437

[bib46] Mackay T. F. C., Stone E. A., and Ayroles J. F., 2009 The genetics of quantitative traits: challenges and prospects. Nat. Rev. Genet. 10: 565–577. 10.1038/nrg2612 19584810

[bib47] Mackay T. F. C., 2014 Epistasis and quantitative traits: using model organisms to study gene-gene interactions. Nat. Rev. Genet. 15: 22–33. 10.1038/nrg3627 24296533 PMC3918431

[bib48] Malaspinas A.-S., 2016 Methods to characterize selective sweeps using time serial samples: an ancient DNA perspective. Mol. Ecol. 25: 24–41. 10.1111/mec.13492 26613371

[bib49] Neuenschwander S., Hospital F., Guillaume F., and Goudet J., 2008 quantiNemo: an individual-based program to simulate quantitative traits with explicit genetic architecture in a dynamic metapopulation. Bioinformatics 24: 1552–1553. 10.1093/bioinformatics/btn219 18450810

[bib50] van Oortmerssen G. A., and Bakker T. C. M., 1981 Artificial selection for short and long attack latencies in wild *Mus musculus domesticus*. Behav. Genet. 11: 115–126. 10.1007/BF01065622 7196726

[bib51] Orr H. A., 1998 The population genetics of adaptation: The distribution of factors fixed during adaptive evolution. Evolution 52: 935–949. 10.1111/j.1558-5646.1998.tb01823.x 28565213

[bib52] Otto S. P., and Jones C. D., 2000 Detecting the undetected: estimating the total number of loci underlying a quantitative trait. Genetics 156: 2093–2107. 11102398 10.1093/genetics/156.4.2093 PMC1461347

[bib53] Parts L., F. Cubillos, J. Warringer, K. Jain, F. Salinas, *et al.*, 2011 Revealing the genetic structure of a trait by sequencing a population under selection. Genome Res. gr.116731.110. 10.1101/gr.116731.110 PMC3129255 21422276

[bib54] Pigeon G., Festa‐Bianchet M., Coltman D. W., and Pelletier F., 2016 Intense selective hunting leads to artificial evolution in horn size. Evol. Appl. 9: 521–530. 10.1111/eva.12358 27099619 PMC4831456

[bib55] Roesti M., Moser D., and Berner D., 2013 Recombination in the threespine stickleback genome—patterns and consequences. Mol. Ecol. 22: 3014–3027. 10.1111/mec.12322 23601112

[bib56] Rubin C.-J., Zody M. C., Eriksson J., Meadows J. R. S., Sherwood E., 2010 Whole-genome resequencing reveals loci under selection during chicken domestication. Nature 464: 587–591. 10.1038/nature08832 20220755

[bib57] Sadowska E. T., Baliga‐Klimczyk K., Chrząścik K. M., and Koteja P., 2008 Laboratory model of adaptive radiation: A selection experiment in the bank vole. Physiol. Biochem. Zool. 81: 627–640. 10.1086/590164 18781839

[bib58] Schlötterer C., Kofler R., Versace E., Tobler R., and Franssen S. U., 2015 Combining experimental evolution with next-generation sequencing: a powerful tool to study adaptation from standing genetic variation. Heredity 114: 431–440. 10.1038/hdy.2014.86 25269380 PMC4815507

[bib59] Seabra S. G., Fragata I., Antunes M. A., Faria G. S., Santos M. A., 2018 Different genomic changes underlie adaptive evolution in populations of contrasting history. Mol. Biol. Evol. 35: 549–563. 10.1093/molbev/msx247 29029198

[bib60] Shao H., Burrage L. C., Sinasac D. S., Hill A. E., Ernest S. R., 2008 Genetic architecture of complex traits: Large phenotypic effects and pervasive epistasis. Proc. Natl. Acad. Sci. USA 105: 19910–19914. 10.1073/pnas.0810388105 19066216 PMC2604967

[bib61] Shifman S., Bell J. T., Copley R. R., Taylor M. S., Williams R. W., 2006 A high-resolution single nucleotide polymorphism genetic map of the mouse genome. PLoS Biol. 4: e395 10.1371/journal.pbio.0040395 17105354 PMC1635748

[bib62] Slatkin M., 1975 Gene glow and selection in a two-locus system. Genetics 81: 787–802. 1213276 10.1093/genetics/81.4.787 PMC1213435

[bib63] Smith J. M., and Haigh J., 1974 The hitch-hiking effect of a favourable gene. Genet. Res. 23: 23–35. 10.1017/S0016672300014634 4407212

[bib64] Stetter M. G., Thornton K., and Ross-Ibarra J., 2018 Genetic architecture and selective sweeps after polygenic adaptation to distant trait optima. PLoS Genet. 14: e1007794 10.1371/journal.pgen.1007794 30452452 PMC6277123

[bib65] Sved J. A., 1971 Linkage disequilibrium and homozygosity of chromosome segments in finite populations. Theor. Popul. Biol. 2: 125–141. 10.1016/0040-5809(71)90011-6 5170716

[bib66] Swain D. P., Sinclair A. F., and Hanson J. M., 2007 Evolutionary response to size-selective mortality in an exploited fish population. Proc. R. Soc. Lond. B Biol. Sci. 274: 1015–1022. 10.1098/rspb.2006.0275 PMC2124474 17264058

[bib67] Tenaillon O., Rodríguez-Verdugo A., Gaut R. L., McDonald P., Bennett A. F., 2012 The molecular diversity of adaptive convergence. Science 335: 457–461. 10.1126/science.1212986 22282810

[bib68] Teotónio H., Estes S., Phillips P. C., and Baer C. F., 2017 Experimental evolution with *Caenorhabditis* nematodes. Genetics 206: 691–716. 10.1534/genetics.115.186288 28592504 PMC5499180

[bib69] Therkildsen N. O., Hemmer-Hansen J., Als T. D., Swain D. P., Morgan M. J., 2013 Microevolution in time and space: SNP analysis of historical DNA reveals dynamic signatures of selection in Atlantic cod. Mol. Ecol. 22: 2424–2440. 10.1111/mec.12260 23551301

[bib70] Therkildsen N. O., Wilder A. P., Conover D. O., Munch S. B., Baumann H., 2019 Contrasting genomic shifts underlie parallel phenotypic evolution in response to fishing. Science 365: 487–490. 10.1126/science.aaw7271 31371613

[bib71] Turner T. L., Stewart A. D., Fields A. T., Rice W. R., and Tarone A. M., 2011 Population-based resequencing of experimentally evolved populations reveals the genetic basis of body size variation in *Drosophila melanogaster*. PLoS Genet. 7: e1001336 10.1371/journal.pgen.1001336 21437274 PMC3060078

[bib72] Uusi-Heikkilä S., Sävilammi T., Leder E., Arlinghaus R., and Primmer C. R., 2017 Rapid, broad-scale gene expression evolution in experimentally harvested fish populations. Mol. Ecol. 26: 3954–3967. 10.1111/mec.14179 28500794

[bib73] Vlachos C., and Kofler R., 2018 MimicrEE2: Genome-wide forward simulations of Evolve and Resequencing studies. PLOS Comput. Biol. 14: e1006413 10.1371/journal.pcbi.1006413 30114186 PMC6112681

[bib74] Walsh B., and Lynch M., 2018 Evolution and Selection of Quantitative Traits, Oxford University Press, Oxford 10.1093/oso/9780198830870.001.0001

[bib75] Wickham H., and RStudio, 2017 tidyverse: Easily install and load the “tidyverse.”

[bib76] Wilke C. O., and RStudio, 2019 cowplot: Streamlined plot theme and plot annotations for “ggplot2.”

[bib77] Wong A. K., Ruhe A. L., Dumont B. L., Robertson K. R., Guerrero G., 2010 A comprehensive linkage map of the dog genome. Genetics 184: 595–605. 10.1534/genetics.109.106831 19966068 PMC2828735

[bib78] Zanini F., and Neher R. A., 2012 FFPopSim: an efficient forward simulation package for the evolution of large populations. Bioinformatics 28: 3332–3333. 10.1093/bioinformatics/bts633 23097421 PMC3519462

[bib79] Zhou D., Udpa N., Gersten M., Visk D. W., Bashir A., 2011 Experimental selection of hypoxia-tolerant *Drosophila melanogaster*. Proc. Natl. Acad. Sci. USA 108: 2349–2354. 10.1073/pnas.1010643108 21262834 PMC3038716

